# Role of S^2−^ ions on the microstructure change and the pitting behaviour of aluminum in saline solution

**DOI:** 10.1038/s41598-019-48503-8

**Published:** 2019-08-19

**Authors:** Mohamed M. EL-Deeb

**Affiliations:** Chemistry Department, Faculty of Science, Beni-Suef University, 62511 Beni-Suef, Egypt

**Keywords:** Chemistry, Materials science

## Abstract

The electrochemical behaviour and the passive film microstructure of aluminum during its exposure to 3.5 wt% NaCl solution in the absence and presence of S^2−^ ions are investigated using potentiodynamic polarization curves, electrochemical impedance spectroscopy measurements, XRD, XRF, SEM and AFM. Electrochemical measurements show that the presence of S^2−^ ions enhances the uniform corrosion of aluminum in NaCl solution, but delay its susceptibility to the pitting corrosion. In addition, EIS analysis illustrate that the formation of more compact and protective passive layer in the presence of S^2−^ ions compared to its rough surface in the absence of S^2−^ ions as evidenced by the lower value of constant phase element (*CPE*) and higher value of phase shift (*N*). Cracks, non- homogenous and open large pits with high degree of roughness are clearly observed on the aluminum surface in the absence of S^2−^ ions, compared to oriented grooves, elongated ridges with the accumulation of the corrosion products inside the pits in the presence of S^2−^ ions. The inhibitory effect of S^2−^ ions for the pitting corrosion of aluminum is interpreted on the basis of the change in its microstructure of the passive film in the absence and presence of S^2−^ions.

## Introduction

Aluminum and its alloys are widely used in industrial applications, because of their good mechanical and industrial properties as well as, their highly corrosion protection^1–4^. Aluminum when immersed in chloride solutions, it will expose to the pitting corrosion, that limits its industrial and marine applications^5^. The pitting corrosion of 7A60 aluminum alloy in 3.5 wt% NaCl solution was investigated using electrochemical impedance spectroscopy, electrochemical noise, scanning electron microscopy and energy dispersive spectrometer^6^. Results revealed that the intense pitting corrosion in 7A60 aluminum alloy is explained to the presence of electrochemical active MgZn_2_, Al_2_MgCu and Mg_2_Si, while the contribution of Al_7_Cu_2_Fe to the pitting corrosion is little. Zhang *et al*.^7^ studied the relationship between the intergranular corrosion and the crystallographic pitting in AA2024-T351 aluminum alloy in 3.5 wt% NaCl solution. It was found that the intergranular corrosion occurs firstly, and the crystallographic pitting initiates from the crevice wall behind the intergranular corrosion front.

Sulphide polluted saline solutions are very aggressive medium for the corrosion of many metals, that used in many industrial installations processing sulfides. Corrosion behaviour of Cu-Al-5Ni alloy in 3.5 wt% NaCl solution containing different concentrations of sulphide ions was studied using electrochemical impedance spectroscopy measurements^8^. Data showed that, increasing the sulpide ion concentrations leads to increase the corrosion rate of this alloy that can be explained on the basis of the presence of Cu_2_S that decrease the protective efficiencies of Cu_2_O film. Dissolution of aluminum in 0.01 M NaOH solution as a function of sulphate, nitrate and sulphide ion concentrations was evaluated using electrochemical noise measurements^9^. It was determined that the corrosion rate of aluminum in the alkaline solution was occurred more seriousness in the presence of these additives, as concluded from the positive shift in the frequency to more highly frequency region. Sherar *et al*.^10^, studied the effect of the sulphide ions on the aerobic corrosion of steel in near neutral saline solution using scanning electron microscopy, energy dispersive X-ray analysis and Raman spectroscopy. They found that the addition of the sulphide ions to the aerobically-exposed surface doubles the corrosion rate compared to its value in the sulphide free solution. Electrochemical performance of Cu, Cu-10Al-10Ni, Cp-Ti and C-steel in 3.5 wt% NaCl containing 2 ppm Na_2_S was studied^11^. Results clarified that the addition of S^2−^, shifted the corrosion potential for all alloys to more negative values except Cp-Ti that be shifted to more positive.

Morphology and microstructure of the passive film play an important role for its mechanical and electrochemical properties^12^. Liu *et al*.^13^ studied the effect of the abrasion on the microstructural and pitting corrosion of 2297Al-Li alloy in borate-buffered 0.001 M NaCl. Data revealed that the pitting corrosion of this alloy was strongly affected by the size, the population density and the area fraction of intermetallic particles as well as its smoother surface has lower pitting ability and better corrosion resistance compared to the rougher surface. In addition, the crystallographic orientations within a microstructure was observed to be considerable effect on pitting corrosion. It was observed the relation between the polarization and pitting behaviors with the surface orientation in pure aluminum and aluminum alloys^14–20^.

Effect of the microstructure on the corrosion behaviour of cast Mg-Al alloys in 5 wt. % NaCl solution saturated with Mg(OH)_2_ was studied^21^. Data showed that the corrosion protection of homogeneous α-phase increases with increasing Al-content, due to the higher chemical stability of α-phase with higher Al contents compared to its stability with lower Al contents. The effect of heat treatment on the electrochemical corrosion and microstructural behaviour of API X70 line pipe steel in sea water containing thiosulphate solutions was evaluated^22^. Data implies that the presence of different phases of ferrite during heat treatment. Polygonal ferrite and fine grained ferrite microstructures formed at 600 °C inhibits the corrosion rate compared to the tempered martensitic microstructure formed at 300 °C. The inhibition effect was explained on to the difference in the diffusion rate of the corrosion product across the fine and coarse grained microstructure. Corrosion protection of PEO coating on extruded Al6Cu alloy in 3.5% wt NaCl was evaluated^23^. The corrosion protection of Al6Cu alloy was enhanced in the presence of PEO coating compared to as-cast alloy due to the change in its microstructure.

Yang *et al*.^24^ studied the effect of sulphide ions on the passivation behaviour of titanium TA2 alloy in simulated seawater solutions. They found that the passive film was composed of TiO_2_ top layer with TiO sub-oxide layer and the highly resistance of Ti substrate was correlated to the presence of TiO_2_ top layer. In addition, the presence of low concentrations of highly amount of TiO_2_. While at highly concentration of S^2−^ ions (>2 mM/L) decreased the corrosion resistance due to the highly fraction of TiOS and TiS_2_ compared to TiO_2_. The influence of sulphide ions on the passive behaviour of super 13Cr martensitic stainless steel in borate/NaCl solution was investigated^25^. Results showed that the addition of sulphide ions increased the values of the corrosion current and shifted the pitting potentials to more noble values. Moreover, the presence of sulphide ions changed the microstructure and the crystallinity of the passive layer.

By far, to our knowledge, little work reported the effect of the microstructure of the passive film on the pitting behaviour of aluminum in sulphide polluted saline solution. Therefore, the goal of our present work is to investigate the influence of the microstructure of the oxide passive film on the uniform and pitting corrosion behaviour of aluminum in 3.5 wt% NaCl solution in the absence and presence of different concentrations of Na_2_S using atomic force microscopy, scanning electron microscopy, X-ray fluorescence, X-ray diffractometer, potentiodynamic polarization and electrochemical impedance spectroscopy techniques.

## Experimental

### Materials

Sodium chloride, sodium sulphide and acetone are provided from Merck Chemical Co. (Germany). Working electrode is made from aluminum rod with the following chemical composition (wt%): 99.57% Al, 0.31% Fe, 0.07% Si, 0.015% Ti%, 0.0016% Zn, 0.0003% Cr, 0.0019% Mg, 0.0021% Mn and 0.0007 Cu.

### Microstructure characterization

The surface roughness of aluminum samples is examined using AFM measurements (Nanoscopic III E controller, Digital Instruments, Santa Barbara, CA). All AFM experiments are performed with soft cantilevers: n^+^- doped Si cantilever from Nanosensors (PPP-CONT-10), *k*_n_ = 0.09 N m^−1^. Scanning electron microscopy is achieved using JSM-6510LA (JEOL, Tokyo, Japan). Surface composition, crystallinity and phase identification of aluminum samples are investigated using X-ray fluorescence (ARL^TM^ Perform’x Sequential XRF spectrometer, Thermo Scientific) and X-ray diffractometer (PANalytical Empyrean, Netherlands).

### Electrochemical measurements

All the electrochemical measurements are carried out using the Potentiostat/Galvanostat (AUTOLAB PGSTAT 128 N), using a standard three-electrode cell with Al (1.0 cm^2^) working, saturated Ag/AgCl reference and Pt sheet (1.0 cm^2^) counter electrodes. NOVA 1.10 software is used to records and fits the electrochemical measurements. Potentiodynamic polarization measurements are achieved in the potential range between −100 to 200 mV vs. *E*_OCP_ values at 30 °C with the scan rate of 1.0 mV s^−1^. Electrochemical impedance spectra at the respective *E*_OCP_ values are recorded using AC signals of amplitude 5 mV peak to peak in the frequency range of 10 kHz to10 MHz. Prior to each experiment, working electrode is polished successively with fine grade emery papers, cleaned with acetone, washed with bi-distilled water and finally dried.

## Results and Discussion

### Electrochemical measurements

#### Polarization measurements

**Figure 1:**
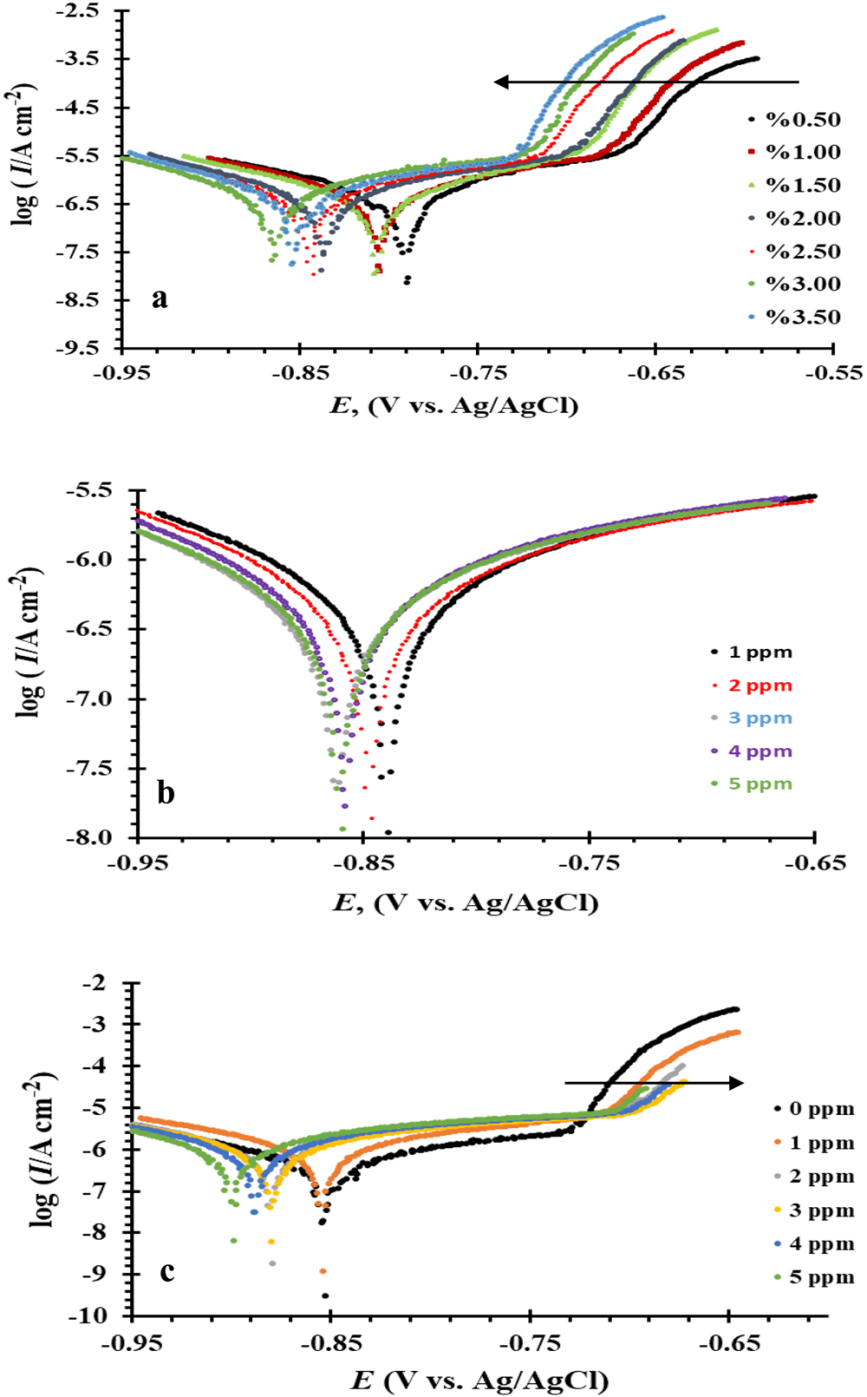
Potentiodynamic polarization curves of Al in (**a**) *x* wt% NaCl, (**b**) *x* ppm Na_2_S and (**c**) 3.5 wt% NaCl + *x* ppm Na_2_S solutions at 30 °C with scan rate of 1.0 mV s^−1^.

Figure 1a represents the potentiodynamic polarization measurements of aluminum electrode in *x* wt% NaCl solution (*x* = 0.5–3.5), while its electrochemical parameters derived from the polarization curves (*E*_corr_*, E*_pit_*, I*_corr_*, β*_a_*, β*_c_) are listed in Table 1. Results show that the presence of active/passive/pitting as the electrochemical behaviour of aluminum for all studied concentrations. It is observed that, increasing the concentrations of Cl^−^ ions, shifts both the corrosion potential (*E*_corr_) and the pitting potential (*E*_pit_) to more negative values as well as increases the values of the corrosion current densities (*I*_corr_) from 0.6 μA cm^−2^ in case of 0.5 wt% NaCl solution to 2.1 μA cm^−2^ for 3.5 wt% NaCl solution. This finding explains that the rate of both uniform and pitting corrosion of aluminum increases as a results of the aggressive attack of Cl^−^ ions. Mechanism of the anodic and cathodic reactions associated with the uniform corrosion of aluminum in Cl^−^ ion solutions based on the previously reported is explained as follows^26–28^:

- 1**Cathodic reaction:**
  1 $${{\rm{O}}}_{2}+2{{\rm{H}}}_{2}{\rm{O}}+2{{\rm{e}}}^{-}\to 4{{{\rm{OH}}}^{-}}_{(\mathrm{sol})}$$
- 2**Anodic reactions:**

2 $${{\rm{Al}}}_{(s)}+{{\rm{3e}}}^{-}\to {{{\rm{Al}}}^{3+}}_{(\mathrm{sol})}$$

3 $${{{\rm{Al}}}^{3+}}_{({\rm{sol}})}+{{\rm{3OH}}}_{(\mathrm{sol})}^{-}\to {\rm{Al}}{({\rm{OH}})}_{3({\rm{s}})}$$

4 $$2{\rm{Al}}{({\rm{OH}})}_{3(s)}\to {{\rm{Al}}}_{{\rm{2}}}{{\rm{O}}}_{3(s)}+3{{\rm{H}}}_{2}{\rm{O}}$$

**Table 1:** Electrochemical kinetic parameters of aluminum in *x* wt% NaCl solutions at 30 °C.

C, (wt %)	*E*_corr_ (V vs. Ag/AgCl)	*I*_corr_ (µA cm^−2^)	*β*_a_ (mV dec^−1^)	*β*_c_ (mV dec^−1^)	*E*_Pit_ (V vs. Ag/AgCl)
0.5	−790	0.6	150.5	158	−676
1.0	−805	0.77	158.5	200	−685
1.5	−806	0.81	158.9	200	−693
2.0	−836	0.82	160.9	218	−698
2.5	−843	0.88	168.4	225	−718
3.0	−852	1.1	184.9	243	−719
3.5	−864	2.1	247.3	314	−729

Moreover, the pitting corrosion of aluminum is explained on the basis of the reaction between the adsorbed Cl^−^ ions with Al^3+^ in the crystal lattice of the dual nature Al_2_O_3_ passive film, that consists of an adherent, compact and stable inner film covered with a porous, less stable outer film to form soluble oxychloride complex as shown in Eq. (5)^29,30^.

5 $${{{\rm{Al}}}^{3+}}_{(\mathrm{crystal}\mathrm{lattice})}+2{{{\rm{Cl}}}^{-}}_{(\mathrm{ads})}+2{{{\rm{OH}}}^{-}}_{(\mathrm{sol})}\to {[{\rm{Al}}{({\rm{OH}})}_{2}{{\rm{Cl}}}_{2}]}^{-}$$

The presence of the adsorbed chloride ions on the Al_2_O_3_ passive film is confirmed using XRF analysis as shown in Table 2. Our results are in a good agreement with Soltis^20^ and Tang *et al*.^31^, who reported that the pitting potential (*E*_pit_) was shifted to more negative values with increasing the concentration of the aggressive ions according to the following relation:

6 $${E}_{{\rm{pit}}}=A-B\,\mathrm{log}\,[{{\rm{Cl}}}^{-}]$$

where *A* and *B* are constants, whilst *A* measures the aggressiveness of the chloride ions at a given concentration. Explanation of this trend can be illustrated as follows:

- At low Cl^−^ concentration, Al^3+^ ions in the crystal lattice of the passive layer prefers to hydrolyze with OH^−^ rather than reacts with the Cl^−^ ions, therefore the growth and the propagation of the pits are decreased^27^.
- At high Cl^−^ concentration, Al^3+^ ions react with Cl^−^ ions producing soluble complex, that accelerates both the nucleation and the propagation of the pits^29,31^.

**Table 2:** XRF analysis of aluminum surface immersed in 3.5 wt% NaCl, 5 ppm Na_2_S and 3.5 wt% NaCl + 5 ppm Na_2_S solutions for 24 hours at 30 °C.

Compound	Al/3.5 wt% NaCl	Al/5 ppm Na_2_S	Al/3.5 wt% NaCl + 5 ppm Na_2_S
Al_2_O_3_	99.42	99.47	99.49
MgO	0.163	0.161	0.161
Fe_2_O_3_	0.128	0.113	0.120
SiO_2_	0.125	0.148	0.125
SO_3_	0.0546	0.0476	0.0183
Na_2_O	0.0389	0.0283	0.0344
Cl	0.0322	—	0.0168
TiO_2_	0.0174	0.0137	0.0167
Ga_2_O_3_	0.0041	0.004	0.0037
CaO	0.0027	0.0021	0.0026
V_2_O_5_	0.0026	0.0027	0.0026
K_2_O	0.0013	0.0011	—
MnO	0.0011	0.0011	—

Figure 1b represents the effect of Na_2_S concentrations on the anodic and cathodic polarization curves of aluminum and the detailed electrochemical parameters are listed in Table 3. The results imply that the uniform corrosion of aluminum increases with increasing the S^2−^ ion concentrations without any sign for pitting corrosion within the studied polarization range. Mechanism of the uniform corrosion of aluminum in S^2−^ solution can be summarized as previously reported^8,32–34^, S^2−^ ions is hydrogenated to give HS^−^, that reacts with aluminum to produces Al_2_S_3_ and finally Al_2_O_3_ according to the following equations:

7 $${{\rm{S}}}^{2-}+{{\rm{H}}}_{2}{\rm{O}}\to {{\rm{HS}}}^{-}+{{\rm{OH}}}^{-}$$

8 $${{\rm{Al}}}^{3+}+{{\rm{HS}}}^{-}+{{\rm{OH}}}^{-}\to {{\rm{Al}}}_{2}{{\rm{S}}}_{3}+{{\rm{H}}}_{2}{\rm{O}}$$

9 $${{\rm{Al}}}_{2}{{\rm{S}}}_{3}+{{\rm{H}}}_{2}{\rm{O}}\to {\rm{Al}}{({\rm{OH}})}_{3}+{{\rm{H}}}_{2}{\rm{S}}$$

10 $$2{\rm{Al}}{({\rm{OH}})}_{3}\to {{\rm{Al}}}_{2}{{\rm{O}}}_{3}+{{\rm{H}}}_{2}{\rm{O}}$$

**Table 3:** Electrochemical kinetic parameters of aluminum in *x* ppm Na_2_S solutions at 30 °C.

C, ppm	*E*_corr_ (V vs. Ag/AgCl)	*I*_corr_ (µA cm^−2^)	*β*_a_ (mV dec^−1^)	*β*_c_ (mV dec^−1^)	*E*_Pit_ (V vs. Ag/AgCl)
1	−839	1.77	366	0.74	—
2	−846	1.92	397	0.84	—
3	−858	2.23	421	0.95	—
4	−861	2.40	506	1.08	—
5	−860	3.15	563	1.34	—

The presence of Al_2_O_3_ as a passive film instead of Al_2_S_3_ is confirmed using XRF analysis (c.f. Table 2). On the other hand, the production of OH^−^ during the hydrogenation of S^2−^ ions as shown in Eq. (7) increases the solution pH, that explains the enhancement of the uniform corrosion rate of aluminum with increasing the concentration of S^2−^ ions.

Addition of different concentrations of Na_2_S (1–5 ppm) to 3.5 wt% NaCl solution, enhances the uniform corrosion of aluminum, compared to 3.5 wt% NaCl and 5 ppm Na_2_S solutions as indicated from the values of both *E*_corr_ and *I*_corr_ as shown in Tables 1, 3 and 4. In contrast, the presence of Na_2_S in NaCl solution inhibits the pitting corrosion of aluminum as can be seen from Fig. 1c. The inhibition effect of S^2−^ ions on the pitting corrosion of aluminum can be seen from the decreasing in the values of the *I*_pit_ and increasing the difference between the values of *E*_corr_ and *E*_pit_ (Δ*E* = *E*_corr_ − *E*_pit_) with increasing S^2−^ ion concentrations (c.f. Fig. 1c).

**Table 4:** Electrochemical kinetic parameters of aluminum in 3.5 wt% NaCl + *x* ppm Na_2_S solutions at 30 °C.

C	*E*_corr_ (V vs. Ag/AgCl)	*I*_corr_ (µA cm^−2^)	*β*_a_ (mV dec^−1^)	*β*_c_ (mV dec^−1^)	*E*_Pit_ (V vs. Ag/AgCl)
3.5/1	−854	2.6	212	0.36	−713
3.5/2	−875	4.4	284	0.67	−705
3.5/3	−879	5.16	331	0.82	−701
3.5/4	−887	5.35	343	0.88	−698
3.5/5	−898	6.35	432	1.17	−696

#### Electrochemical impedance spectroscopy measurements

**Figure 2:**
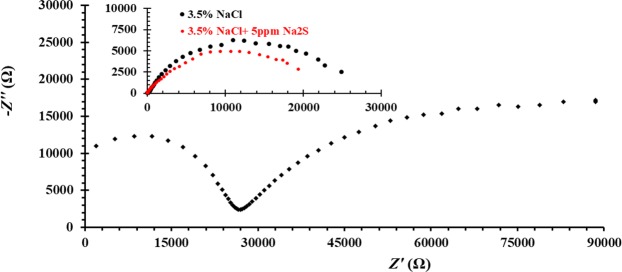
Nyquist plot of aluminum in 5 ppm Na_2_S solution at *E*_OCP._ The inset represents the Nequist plots of aluminum in 3.5 wt% NaCl and in 3.5 wt% NaCl + 5 ppm Na_2_S solutions at *E*_OCP._

Electrochemical impedance spectroscopy measurements are performed to evaluate the Al/electrolyte interface with different electrolyte composition. Figure 2 represents the Nyquest plots of aluminum surface recorded at *E*_OCP_ in the presence of 3.5 wt% NaCl, 5 ppm Na_2_S and mixture of 3.5 wt% NaCl + 5 ppm Na_2_S solutions. Results point out that the presence of two consecutive capacitive semicircles in 5 ppm Na_2_S solutions, that have been interpreted to the dual nature of Al_2_O_3_ passive film including an inner compact layer covered with outer porous layer^35^. Whereas a single semicircle is observed in both 3.5 wt% NaCl and 3.5 wt% NaCl + 5 ppm Na_2_S solutions. This observation has been explained as a results of the adsorption of Cl^−^ ions on the Al_2_O_3_ passive layer, which reacts with Al^3+^ in its crystal lattice, forming soluble oxychloride complex as discussed before, leads to the dissolution of the outer porous layer. Also, the deviations of Nyquest plots in cases of 3.5 wt% NaCl and 3.5 wt% NaCl + 5 ppm Na_2_S solutions from a perfect circular shape refers to frequency dispersion of interfacial impedance arising from the inhomogeneity of the electrode surface due to roughness phenomena^36^. On the other hand, the enhancement in the uniform corrosion of aluminum in 3.5 wt% NaCl + 5 ppm Na_2_S solution compared to 3.5 wt% NaCl and 5 ppm Na_2_S solutions is observed from the semicircle diameters as shown in the Fig. 2.

**Figure 3:**
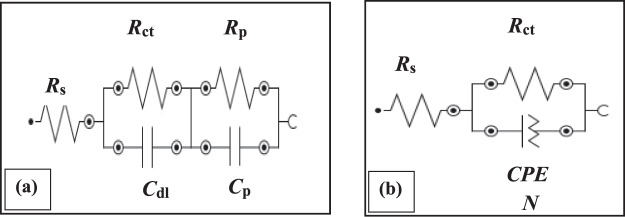
Electrical equivalent circuit model for aluminum/electrolyte interface in (**a**) 5 ppm Na_2_S solution [R(RC)/(RC)] and (**b**) 3.5 wt% NaCl and 3.5 wt% NaCl + 5 ppm Na_2_S solutions [R(RQ)].

Figure 3 represents the fitted electrochemical equivalent circuit as a function in 3.5 wt% NaCl, 5 ppm Na_2_S and 3.5 wt% NaCl + 5 ppm Na_2_S solutions. It can be seen that the Nequest plot of aluminum in 5 ppm Na_2_S solution shows [*R*(*RC*)/(*RC*)] equivalent circuit with two time constants, that agreed with the dual nature of the Al_2_O_3_ passive film formed in the Al/electrolyte interface. Whereas [*R*(*RQ*)] equivalent circuit with one-time constant is fitted to the experimental data obtained in 3.5 wt% NaCl and 3.5 wt% NaCl + 5 ppm Na_2_S solutions due to the dissolution of the outer Al_2_O_3_ passive layer under the influence of its attack with the adsorbed Cl^−^ ions. Moreover, the replacement of the double layer capacitance (*C*_dl_) in the [*R*(*RC*)/(*RC*)] equivalent circuit with constant phase element (*CPE*) and phase shift (*N*) in [*R*(*RQ*)] equivalent circuit explains the dispersion effect and the degree of heterogeneity resulted from the microscopic roughness of aluminum surface due to pitting corrosion^37–39^. The highest value of *N* accompanied with smallest value of *CPE* for aluminum in 3.5 wt% NaCl + 5 ppm Na_2_S solution (*N* = 0.56 and *CPE* = 54.9 µMho) compared to their values in 3.5 wt% NaCl solution (*N* = 0.52 and *CPE* = 66.1 µMho) is calculated from the fitted experimental measurements using Boukamp model^40,41^. This means that, Al/electrolyte interface in 3.5 wt% NaCl + 5 ppm Na_2_S solution behaves as a more ideal capacitive rather than that in 3.5 wt% NaCl, which illustrates the role of the S^2−^ ions in decreasing the roughness and increasing the homogeneity of the aluminum surface.

It is concluded from the electrochemical measurements that, even the presence of S^2−^ ions in NaCl solution enhances the uniform corrosion of aluminum but delay its susceptibility to pitting corrosion. This finding can explained on the bases of the accumulation of the corrosion products on the aluminum surface that increases the stability and the protectionist of Al_2_O_3_ passive layer and consequently, inhibits the rate of the pitting corrosion.

### Microstructures of the aluminum surface

**Figure 4:**
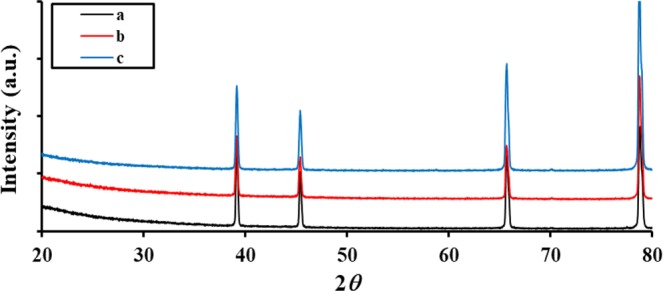
XRD patterns of the as-received aluminum surface immersed in (**a**) 3.5 wt% NaCl, (**b**) 5 ppm Na_2_S and (**c**) 3.5 wt% NaCl + 5 ppm Na_2_S solutions for 24 hours at 30 °C.

**Figure 5:**
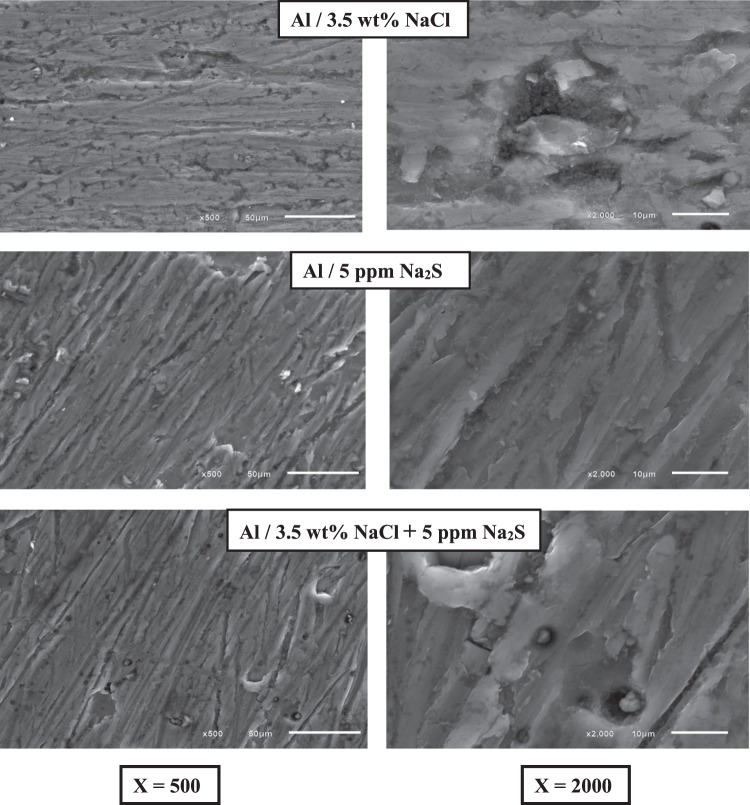
SEM images of the as-received aluminum surface immersed in 3.5 wt% NaCl, 5 ppm Na_2_S and 3.5 wt% NaCl + 5 ppm Na_2_S solutions for 24 hours at 30 °C.

Figure 4 and Table 2 represents the surface composition, crystallinity and phase identification of as received aluminum surface immersed in 3.5 wt% NaCl, 5 ppm Na_2_S and 3.5 wt% NaCl + 5 ppm Na_2_S using X-ray fluorescence and X-Ray Diffraction analysis. Data shows that the phase composition of the passive layer is Al_2_O_3_ in all studied solutions, with 3.23, 0.154 and 2.127 µm average crystal size formed in 3.5 wt% NaCl, 5 ppm Na_2_S and 3.5 wt% NaCl + 5 ppm Na_2_S solutions respectively. SEM images for as received aluminum surface immersed in 3.5 wt% NaCl, 5 ppm Na_2_S and 3.5 wt% NaCl + 5 ppm Na_2_S solutions are represented in Fig. 5. Cracks, non- homogenous and open large pits with high degree of roughness are clearly observed on the aluminum surface exposed to 3.5 wt% NaCl solution in the absence of S^2−^ ions, compared to oriented grooves, elongated ridges with the accumulation of the corrosion products inside the pits in the presence of S^2−^ ions. Whilst, an intact surface with no cracks or pits are observed in case of 5 ppm Na_2_S free Cl^−^ ions solution.

**Figure 6:**
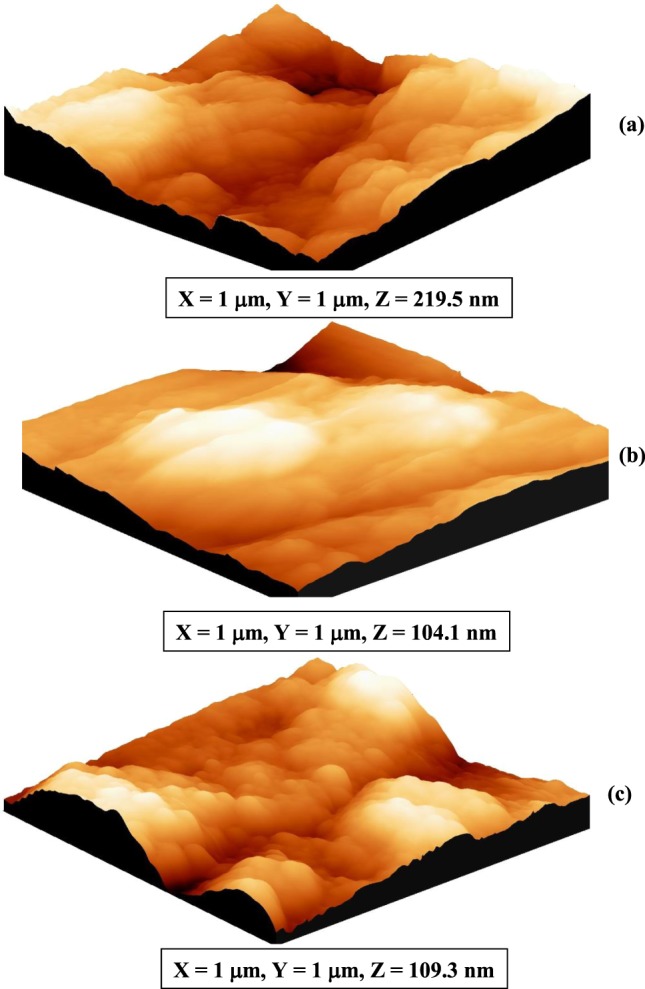
AFM images of the as-received aluminum surface immersed in (**a**) 3.5 wt% NaCl, (**b**) 5 ppm Na_2_S and (**c**) 3.5 wt% NaCl + 5 ppm Na_2_S solutions for 24 hours at 30 °C.

Topography of the aluminum surface immersed in 3.5 wt% NaCl, 5 ppm Na_2_S and 3.5 wt% NaCl + 5 ppm Na_2_S solutions is represented in Fig. 6, while its morphological parameters (*RMS* and *R*_a_) are tabulated in Table 5. Data shows that the extensive pitting corrosion throughout the aluminum surface exposed to 3.5 wt% NaCl solution in the absence of S^2−^ ions is indicated by the large values of both root mean square roughness (*RMS*) and average roughness (*R*_a_) compared to their values in the presence of S^2−^ ions. This behaviour is in a good agreement with the electrochemical measurements.

**Table 5:** AFM analysis results for aluminum surface immersed in 3.5 wt% NaCl, 5 ppm Na_2_S and 3.5 wt% NaCl + 5 ppm Na2S solutions for 24 hours at 30 °C.

Sample	RMS (μm)	R_a_ (μm)
Al/3.5 wt% NaCl	0.355	0.343
Al/5 ppm Na_2_S	0.204	0.161
Al/3.5 wt% NaCl + 5 ppm Na_2_S	0.325	0.324

**Figure 7:**
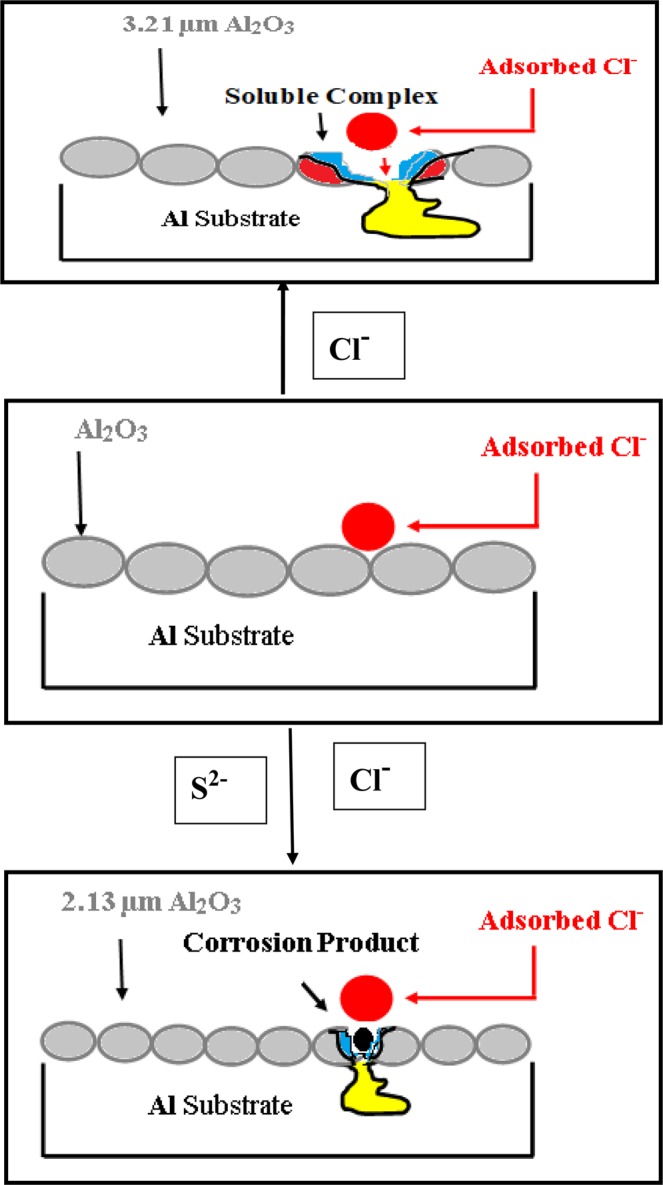
Schematic diagram represented the effect of S^2−^ ions on the pitting corrosion of aluminum.

The enhancement in both the roughness and the homogeneity of aluminum surface in the presence of S^2−^ ions is due to the formation of smoother, fine crystalline sized Al_2_O_3_ passive layer that accumulates inside the pits and inhibits their propagation, therefore decreases the susceptibility of the aluminum surface towards aggressive pitting corrosion in NaCl solution as schematically represented in Fig. 7.

## Conclusion

It can be concluded that the electrochemical behaviour and the microstructure of the passive film of aluminum during its exposure to 3.5 wt% NaCl solution is strongly affected by the presence of S^2−^ ions. Increasing the concentrations of S^2−^ ions in 3.5 wt% NaCl solution increases the pH values of the saline solution, thus increasing both the anodic reaction of Al dissolution and the dissolution kinetics of Al_2_O_3_ passive layer resulting in an increase of the uniform corrosion. At the same time, the lower kinetic formation of smoother, compact, fine crystalline sized Al_2_O_3_ inhibits the pitting corrosion. The inhibitory effect is interpreted on the basis that the smooth, fine crystalline surface of Al_2_O_3_ increases its stability and decreases the aggressiveness of the Cl^−^ ions. Consequently, decreases the rate of the initiation and the growth of the stable pits.

## Data Availability

The dataset generated or analyzed during the current study are available from the corresponding author upon request.

## References

[1] Abdallah M, Kamar EM, Eid S, El-Etre AY (2016). Animal glue as green inhibitor for corrosion of aluminum and aluminum-silicon alloys in sodium hydroxide solutions. J Mol Liq.

[2] Sayyah SM, El-Deeb MM, Abd El-Rehim SS, Ghanem RA, Mohamed SM (2014). Experimental and theoretical evaluation on the effect of the terminal side chain of a polymeric surfactant on the inhibition efficiency of aluminum corrosion in acid medium. Port Electrochim Acta.

[3] Hurlen T, Lian H, Ogegrd OS, Valand TV (1984). Corrosion and passive behaviour of aluminium in weakly acid solution. Electrochim Acta.

[4] El-Deeb MM, Ads EN, Humaidi JR (2018). Evaluation of the modified extracted lignin from wheat straw as corrosion inhibitors for aluminum in alkaline solution. Int J Electrochem Sci.

[5] El-Deeb MM, Abdel-Shafi NS, Shamroukh AH (2018). Electrochemical, DFT and Mont Carlo simulations studies to evaluate the inhibition effect of novel pyridazine derivatives on iron pitting corrosion in 3.5% NaCl. Int J Electrochem Sci.

[6] Wang X-H, Wang J-H, FU C-W (2014). Characterization of pitting corrosion of 7A60 aluminum alloy by EN and EIS techniques. T Nonferr Metal Soc.

[7] Zhang X, Zhou X, Hashimoto T, Liu B (2017). Localized corrosion in AA2024-T351 aluminum alloy: Transition from intergranular corrosion to crystallographic pitting. Mater Charact.

[8] El-Sayed NH, El-Rabiei MM (2014). Effect of sulphide pollution on the stability of Cu-Al-5Ni alloy in 3.5% NaCl solution. Egypt. J Petrol.

[9] Na K-H, Pyun S-I (2007). Electrochemical noise analysis of corrosion of pure aluminum in alkaline solution in the presence of SO_4_^2−^ ion, NO_3_^−^ ion and Na_2_S additives. Electrochim Acta.

[10] Sherar BW, Keech PG, Shoesmith DW (2013). The effect of sulphide on the aerobic corrosion of carbon steel in near-neutral pH saline solution. Corros Sci.

[11] Nady H, El-Rabiei MM, Samy M (2017). Corrosion behaviour and electrochemical properties of carbon steel, commercial pure titanium, copper and copper-aluminum-nickel alloy in 3.5% sodium chloride containing sulphide ions. Egypt J Petrol.

[12] Guo Q, Liu J, Yu M, Li S (2015). Effect of passive film on mechanical properties of martensitic stainless steel 15-5PH in a neutral NaCl solution. Appl Surf Sci.

[13] Liu J, Zhao K, Yu M, Li S (2018). Effect of surface abrasion on pitting corrosion of Al-Li alloy. Corros Sci.

[14] Brewick PT (2017). Microstructure-sensitive modeling of pitting corrosion: Effect of the crystallographic orientation. Corros Sci.

[15] Yasuda M, Weinberg F, Tromans D (1990). Pitting corrosion of Al and Al-Cu single crystals. J Electrochem Soc.

[16] Treacy GM, Breslin CB (1998). Electrochemical studies on single-crystal aluminum surfaces. Electrochim Acta.

[17] Davis BW, Moran PJ, Natishan PM (2000). Metastable pitting behavior of aluminum single crystals. Corros Sci.

[18] Seo JH, Ryu JH, Lee DN (2003). Formation of crystallographic etch pits during AC etching of aluminum. J. Electrochem Soc.

[19] Koroleva E.V, Thompson G.E, Skeldon P, Noble B (2007). Crystallographic dissolution of high purity aluminium. Proceedings of the Royal Society A: Mathematical, Physical and Engineering Sciences.

[20] Soltis J (2015). Passivity breakdown, pit initiation and propagation of pits in metallic materials Review. Corros Sci.

[21] Michael G, Andreas L, Robert S, Sannakaisa V (2019). Influence of the microstructure on the corrosion behaviour of cast Mg-Al alloys. Corros Sci.

[22] Sharm L, Chhibber R (2019). Microstructure evolution and electrochemical corrosion behaviour of API X70 linepipe steel in different environments. Int J Pres Vis Pip.

[23] Zhu L (2019). Microstructure and corrosion resistance of the PEO coating on extruded Al6Cu alloy. Surf Coat Technol.

[24] Yang X, Du C, Wan H, Liu Z, Li X (2018). Influence of sulphides on the passivation behaviour of titanium alloy TA2 in simulated seawater environments. Appl Surf Sci.

[25] Lei X (2019). Passivity of martensitic stainless steel in borate buffer solution: Influence of sulphide ion. Appl Surf Sci.

[26] Amin MA (2010). A newly synthesized glycine derivative to control uniform and pitting corrosion processes of Al induced by SCN^−^ anions – Chemical, electrochemical and morphological studies. Corros Sci.

[27] Zhang K (2018). Inhibitory effect of konjac glucomanan on pitting corrosion of AA5052 aluminum alloy in NaCl solution. J Colloid Interf Sci.

[28] Wang D (2015). Electrochemical and DFT studies of quinoline derivatives on corrosion inhibition of AA5052 aluminum alloy in NaCl solution. Appl Surf Sci.

[29] Sherif E, El-Danaf E, Soliman M, Almajid A (2012). Corrosion passivation in natural seawater of aluminum alloy 1050 processed by equal-channel-angular-press. Int J Electrochem Sci.

[30] Brett C, Gomes I, Martins J (1994). The electrochemical behaviour and corrosion of aluminum in chloride media. The effect of inhibitor anion. Corros Sci.

[31] Tang Y (2014). The metastable pitting potential and its relation to the pitting potential for four materials in chloride solutions. Corros Sci.

[32] Guan F (2017). Influence of sulfate-reducing bacteria on the corrosion behavior of 5052 aluminum alloy. Surf Coat Technol.

[33] Rockel, M. B., Schedlitzky, D. & Bender, R. Aluminum alloys, Corrosion Handbook. *Wiley-VCH Vertag GmbH&Co. KGaA* (2008).

[34] Wiberg, N. & Holleman, A. F. Inorganic Chemistry. Academic Press: San Diego (2001).

[35] Datta J (2008). Role of Cl^−^ and NO_3_^−^ ions on the corrosion behaviour of 20% SiCp reinforced 6061-Al metal matrix composite: A correlation between electrochemical studies and atomic force microscopy. Corros Sci.

[36] Bouyanzer A, Hammouti B, Majidi L (2006). Pennyroyal oil from Mentha pulegium as corrosion inhibitor for steel in 1 M HCl. Mat Lett.

[37] Jüttner K (1990). Electrochemical impedance spectroscopy (EIS) of corrosion processes on inhomogeneous surfaces. Electrochim Acta.

[38] Peng GS, Chen KH, Fang HC, Chao H, Chen SY (2010). EIS study on pitting corrosion of 7150 aluminum alloy in sodium chloride and hydrochloric acid solution. Mater Corros.

[39] Cao, C & Zhang, J. Electrochemical impedance spectroscopy introduction. Science Press, Beijing (2002).

[40] Boukamp BA (1986). A nonlinear least squares fit procedure for analysis of immittance data of electrochemical systems. Solid State Ionics.

[41] El-Deeb MM, Alshammari HM, Abdel-Azeim S (2017). Effect of ortho-substituted aniline on the corrosion protection of aluminum in 2 mol/L H_2_SO_4_ solution. Can J Chem.

